# Vapor Pressures of Platinum, Iridium, and Rhodium

**DOI:** 10.6028/jres.065A.034

**Published:** 1961-08-01

**Authors:** R. F. Hampson, R. F. Walker

## Abstract

The vapor pressures of platinum, iridium, and rhodium have been measured using a microbalance technique based on the Langmuir method. Heats of sublimation at 298 °K were calculated with the aid of free energy functions. The least square lines for the vapor pressure data, the heats of sublimation, and the normal boiling points obtained were as follows:

1. Platinum:
  $$\begin{array}{l} \;\mathrm{Log}\;P_{\text{atm}}=6.761-\frac{27,575}{T}\;(1,916\;\text{to}\;2,042{}^{\circ}K)\\ \Delta H_{s}^{{}^{\circ}}(298)=134.9\pm 1.0\;\text{kcal}/\text{mole}\\ \;\text{bp}=4,100\pm 100{}^{\circ}K \end{array}$$
2. Iridium:
  $$\begin{array}{l} \;\mathrm{Log}\;P_{\text{atm}}=7.139-\frac{33,337}{T}\;(1,986\;\text{to}\;2,260{}^{\circ}K)\\ \Delta H_{s}^{{}^{\circ}}(298)=159.9\pm 2.0\;\text{kcal}/\text{mole}\\ \;\text{bp}=4,800\pm 100{}^{\circ}K \end{array}$$
3. Rhodium:
  $$\begin{array}{l} \;\mathrm{Log}\;P_{\text{atm}}=6.894-\frac{27,276}{T}\;(1,709\;\text{to}\;2,075{}^{\circ}K)\\ \Delta H_{s}^{{}^{\circ}}(298)=132.5\pm 2.0\;\text{kcal}/\text{mole}\\ \;\text{bp}=4,000\pm 100{}^{\circ}K. \end{array}$$

The indicated uncertainties are estimates of the overall limits of error. The value of the gas constant *R* used in the calculation of $\Delta H_{s}^{{}^{\circ}}$ is 1.98726 cal/degree mole.

## 1. Introduction

Systematic measurements of the vapor pressure of iridium and rhodium over a wide temperature range have not been published previously. A summary of previous estimates or observations of their normal boiling points and values of their heats of sublimation, $\Delta H_{s}^{{}^{\circ}}(298)$, is given in table 1.

The vapor pressure of platinum was calculated by Langmuir and Mackay from measurements of the rate of sublimation in vacuum [5].^1^ Subsequently, Jones, Langmuir, and Mackay [6] adjusted these data in accordance with a revised temperature scale. Stull and Sinke used tabulated thermal functions and the adjusted data to calculate a $\Delta H_{s}^{{}^{\circ}}(298)$ of 134.8 kcal/mole and a boiling point of 4,100 °K [4].

Simultaneously with the present investigation, Dreger and Margrave [7] at the University of Wisconsin and Hasapis, Panish and Rosen [8] at the AVCO Corp., Wilmington, Mass., have been measuring vapor pressures of the pertinent substances.

Dreger and Margrave measured rates of sublimation of iridium, rhodium, and platinum samples suspended in a graphite tube furnace. Their data for iridium and rhodium as reported are subject to an additional correction and will not, therefore, be quoted in detail. The data on platinum have just been published [9]. Over the temperature range of 1,571 to 1,786 °K, the vapor pressure of the solid platinum was given by: $\log\;P_{\text{mm}}=10.362-\frac{29,100}{T},$ which leads to a normal boiling point of 4,100±100 °K. The mean $\Delta H_{s}^{{}^{\circ}}(298)$ was 135.2 kcal/mole, with a mean deviation of 0.85 kcal/mole.

Hasapis, Panish, and Rosen have given preliminary data on the vapor pressure of iridium over the temperature range 2,100 to 2,600 °K. A thoria effusion cell was used for these measurements, and the slope of a Clausius-Clapeyron plot of the data yielded a tentative $\Delta H_{s}^{{}^{\circ}}$ of 155±5 kcal/mole over this range of measurement. More recently, Panish and Reif have made measurements of the rates of sublimation of iridium and have used the effusion method to obtain rhodium vapor pressures [10]. For iridium they give $\Delta H_{s}^{{}^{\circ}}(298)=158.4\;\text{kcal}/\text{mole}$ with a mean deviation of 0.4 kcal/mole and an estimated boiling point of 4,800 °K. The corresponding values for rhodium are 132.8 and 0.3 kcal/mole and 3,980 °K.

In all the foregoing measurements it was assumed that the substances sublime to monatomic gaseous species. It was further assumed that the coefficients of sublimation are unity, when calculating vapor pressures from the rate measurements. The lack of agreement among different observations or measurements may be broadly attributed to differences and uncertainties in the experimental techniques.

## 2. Experimental Technique

The experimental technique used in the current investigation was based on the Langmuir method for determining the equilibrium vapor pressure (*P*) from measurements of the rate of sublimation (*m*) at absolute temperature *T*, in accordance with the equation:

$$P=\left(\frac{m}{\alpha }\right)\sqrt{\frac{2\pi RT}{M}}.$$

The rate of sublimation was measured in terms of the mass of material leaving unit area of the subliming surface per unit of time, in vacuum. A value of unity has been adopted for the coefficient of sublimation (*α*) for all calculations of vapor pressures. However, as discussed later, it is probable that this represented an upper limiting value that was not always appropriate under the experimental conditions.^2^ It has also been assumed that the appropriate value of the molecular weight of the vapor species (*M*) is that of the monomeric species. Actual vapor pressures would be somewhat lower than calculated, if polymeric vapor species were involved.

### 2.1. Apparatus

The apparatus used has been described in some detail previously [11, 12]. Briefly, the sample was suspended from an equi-arm, quartz beam microbalance into a water-cooled, glass furnace chamber and heated by induction. The microbalance had a sensitivity of about 1 *μ*g. The sample was attached to the balance by drilling a narrow (0.010 in. diam) hole through the sample and looping a short length of fine (0.002 in. diam) wire of the same substance through the hole. The loop was then placed over a hook on the balance suspension, which consisted of a chain of 0.010 in. diam sapphire rods. The presence of the hole and the loop (which was not heated significantly by the induction field) was ignored when calculating the effective surface area of the load.

The radiofrequency induction field from an external coil was concentrated in the region of the sample with the aid of a water-cooled, copper concentrator held within the furnace chamber. The concentrator had a vertical, uniform bore, ½ in. diam × 1½ in. long, into which the sample was suspended. Water-cooled copper, therefore, enclosed the sample throughout each run, except for the open ends of the bore.

The furnace chamber and the microbalance housing were continuously pumped throughout each run with a liquid-nitrogen-trapped, oil diffusion pump. Pressures within the system were measured with an ionization gauge attached at the vacuum port of the furnace chamber.

### 2.2. Samples

The samples consisted of short rods, approximately ¾ in. long × 0.085 in. diam, and were suspended with their axes vertical. The surface areas at the run temperatures were determined by correcting the room temperature areas of about 1.3 cm^2^ using literature values for the thermal expansion coefficients. No corrections were applied for the slight change in surface area of each sample due to sublimation. Assuming each sample sublimed uniformly over its surface area, the error introduced by neglecting the change in surface area was less than 0.3 percent.

The purity of the samples was estimated from general qualitative spectrochemical analyses. The iridium sample was estimated to be at least 99.99 percent pure, the only major impurity being Rh at the 0.001 to 0.01 percent level; traces of Al, Fe, and Pd, in amounts less than 0.001 percent, were the only other elements detected in the sample. Estimated impurities in the rhodium sample were: Fe, Ir, Pd, Pt, 0.1 to 1 percent; Al, Ca, Cr, Cu, Ni, Ru, 0.01 to 0.1 percent; and B, Si, Ti 0.001 to 0.01 percent. It may have contained less than 98 percent rhodium. Pd, Ir, Rh, at the 0.01 to 0.1 percent level, were the major impurities in the platinum sample. Cu and Fe were each estimated to be in the range 0.001 to 0.01 percent and Ag as less than 0.001 percent. The platinum sample was, therefore, probably better than 99.7 percent purity.

### 2.3. Procedure

The microbalance was used as a deflection instrument, deflections of its beam being directly proportional to changes in mass of the sample. Beam deflections were measured with a cathetometer readable to 1 *μ* displacement. The microbalance was calibrated, with each sample *in situ*, using Class M microbalance weights previously calibrated by the Mass Section of NBS.

Interaction between the rf field and the sample caused the balance to be deflected increasingly as the output of the rf generator was increased. By starting with the sample close to the center of the bore in the concentrator in a vertical direction, the deflection could be reduced to a minimum; however, a continuous record of the change in mass of the sample was still not obtainable with reliability. The balance was, therefore, first read with the sample at room temperature. The sample was then heated rapidly according to a predetermined schedule, held at constant temperature for a given period of time, then cooled rapidly. When the sample had returned to room temperature, the balance was then read again to obtain the total mass change during the course of each run.

The procedure adopted raises a question as to the extent to which mass changes occurring during heating and cooling contributed to the total observed mass change. The variation of vapor pressures with temperature was such that the mass-loss rates occurring 50 to 100 °K below the constant temperature were generally of the order of 1 percent of that occurring at the constant temperature. On heating, the last 300 to 400 °K of the temperature rise was accomplished in less than 15 sec. The relatively low-heat content of the hot system also resulted in an even more rapid rate of cooling over the first few hundred degrees when the power was turned off. Thus, even though a sample was held at a constant temperature for a period as short as ½ min, the observed mass change could be attributed entirely to the constant temperature period without introducing gross errors. In fact, no systematic decrease in the scatter of the data was detected as the length of runs increased from ½ min to 3 hr.

For each substance the duration of the runs was increased from about 1 min at the highest temperatures to about 2 or 3 hr at the lowest temperatures, to yield weight losses of about 100 micrograms. The uncertainty in the measurement of the weight loss during each run was less than ±2 percent.

No special procedure was used to clean the samples after handling. It was assumed that outgassing and cleanup of the surface would occur during the first few runs and that, if there were significant surface contamination, the data would appear to be sufficiently anomalous to justify their rejection. Samples were not normally exposed to atmospheric pressure between runs. Due consideration was given to possible recontamination of the surface whenever it became necessary to open up the evacuated system to add a tare weight to the balance or to examine the sample.

Pressures within the system were in the range 2×10^−6^ to 8×10^−5^ mm Hg throughout each run. Due to outgassing, pressures tended to rise to the upper portion of this range during the first runs after the system was exposed to atmosphere pressure. Subsequent runs yielded successively lower maximum pressures. No trends in the data with changes of pressure in the system were detected.

### 2.4. Temperature Measurement and Control

Brightness temperatures were measured by sighting with an optical pyrometer having an effective wavelength of about 0.660 to 0.665 *μ*, about midway down the length of the sample. Pyrometer readings were corrected by comparing the scale of the pyrometer with that of a similar pyrometer calibrated on the International Temperature Scale by the Temperature Physics Section of NBS. A correction was then applied for the absorption by a window in the furnace chamber.

The angle of sighting was 75° to the normal; separate calibrations were, therefore, also obtained for each sample to correct brightness temperatures for the angle of sighting. These calibrations were undertaken as the samples were heated in air. Under these conditions the rhodium sample appeared to acquire and retain an oxide coating throughout the pertinent temperature range, and the sign of the temperature correction was characteristic of a nonmetal rather than of a metal. Consequently, the corrections obtained for the platinum sample were also applied to the rhodium sample, and an additional uncertainty of ±5 °C was assigned to the final brightness temperatures of the rhodium.

Corrected brightness temperatures were converted to thermodynamic temperatures with the aid of literature values for the normal spectral emissivities, ϵλ, at the wavelength, λ, given by the respective authors.

Stephens [13] gives values for the emissivity of platinum for λ=0.660 *μ.* An extrapolation of his data vields values of *ϵ*_λ_ increasing linearly from 0.293 at 1,700 °K to 0.300 at 2,050 °K. The data of Worthing [14] have approximately the same temperature dependence, but are significantly higher, yielding (when extrapolated) *ϵ*_λ_ rising from 0.307 at 1,700 °K to 0.315 at 2,050 °K (λ=0.665 *μ*). A check on the appropriate values to be used in the present work was made by observing the brightness temperature at the onset of surface melting of the platinum sample under the experimental conditions. The results of the observations are summarized in table 2. Using λ=0.660 *μ*, a value of *ϵ*_λ_=0.308 was calculated from these observations and the known melting point of platinum. A temperature dependence was assigned to this value, yielding a normal emissivity rising linearly from 0.304 at 1,850 °K to 0.308 at 2,050 °K. These values are about the mean of those given by Stephens and Worthing, and have been used in the present work.

The emissivities of iridium and rhodium are not well established; in particular, no data for the dependence of their emissivities on temperature are available. An additional uncertainty, upon which it is not possible to place close limits, is, therefore, involved in the temperatures of these substances. Goldwater and Danforth [15] found the mean spectral emissivity of iridium to be 0.33 (λ=0.65 *μ*) for measurements over the 1,000 to 1,750 °C range. Whitney [16] observed the emissivity of rhodium to increase with temperature over the range 1,700 to 2,200 °K, for a sample subjected to only limited heat treatment. However, after 700 hr of heat treatment a constant value of *ϵ*_λ_=0.242 (λ=0.667 *μ*) was obtained for the entire range of 1,300 to 2,000 °K.

The foregoing values of *ϵ*_λ_ have been used to obtain the thermodynamic temperatures of iridium and rhodium in the present work.

Constant temperatures were maintained to within ±5 °C or better by manual control of the output of the rf generator. No significant temperature gradients could be observed down the length of samples. Sightings on the top or upper end of the rod as suspended gave temperatures which were consistent with those observed down the length when the different angle of sighting was taken into consideration (the angle was about 15° to the normal for the top of the sample).

## 3. Results and Discussion

### 3.1. Platinum

In table 3 are given the vapor pressures calculated from measurements of the rate of mass loss of platinum and the corresponding values of $\Delta H_{s}^{{}^{\circ}}(298)$ calculated using the free energy functions of Stull and Sinke [4].

An original purpose of the measurements with platinum was to check the apparatus and technique using a substance whose vapor pressures had been previously measured. It was unfortunate, therefore, that the data obtained for this substance showed the least internal consistency. Several runs yielded unexpectedly low rates of sublimation. As indicated in table 3, some of the rates were too low to detect significant mass losses during the period of the runs; other low rates were measurable, but scattered and have been rejected as being clearly inconsistent with the bulk of the data. The rejected data have been marked with an asterisk in table 3. As far as could be ascertained, the anomalous data were not due to any misbehavior of the balance or of other components of the apparatus. They occurred below some critical temperature, which apparently increased with more extensive heat-treatment at higher temperatures. During the course of one run in which the sample was vaporizing at a very low rate, the temperature was raised rapidly just a few degrees, and the “normal” rate of sublimation was immediately restored for the remainder of the run.

It has not yet been possible to identify the cause of the low rates of sublimation. The two most likely possibilities are contamination of the surface of the sample either from its surroundings or by migration of impurities to the surface of the sample, and significant changes in the crystal structure of the sample with progressive heat treatment. Nevertheless, the results suggest that platinum may acquire a coefficient of sublimation considerably less than unity under the experimental conditions.

**Figure 1:**
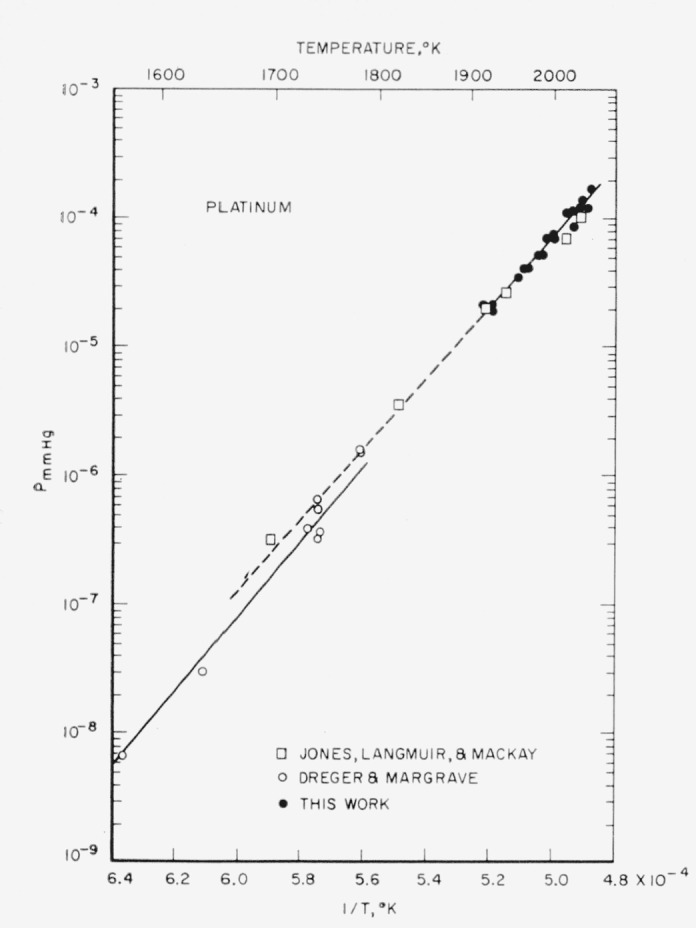
Vapor pressure of platinum versus reciprocal of absolute temperature, for different investigators. The least-squares line representing this work is shown extrapolated with a broken line. The other line is that given by Dreger and Margrave.

Apart from the anomalous data discussed, the remainder of the data had sufficient internal consistency to justify their acceptance. These data are compared with the results of Jones, Langmuir, and Mackay, and of Dreger and Margrave in figure 1. The first two runs shown in table 3 have been rejected because they were the initial runs, and because the values of $\Delta H_{s}^{{}^{\circ}}(298)$ differed significantly from the mean of the accepted runs. Nevertheless, they yield values of $\Delta H_{s}^{{}^{\circ}}(298)$ which are within the range of those of Dreger and Margrave, obtained in the same low temperature region. Furthermore, their data also have more than the usual amount of scatter in this region. Using free-energy functions, the data of Jones et al., yield heats of sublimation which show a slight increase with temperature, from which we calculate a mean $\Delta H_{s}^{{}^{\circ}}(298)$ of 134.6 kcal/mole. Nevertheless, it is interesting to note that the three sets of measurements lead to mean heats which agree within the limits of experimental error. The overall limits of error of the absolute value of $\Delta H_{s}^{{}^{\circ}}(298)$ were estimated by taking into account the scatter of the data, the uncertainty in the temperature (±10° K), and the uncertainty in the weight-loss measurements. No allowance was made for the uncertainty in the free-energy functions or in the sublimation coefficient. The overall limits of error of the absolute value were estimated to be ±1.0 kcal/mole. The corresponding overall limits of error of the vapor pressures are about ±25 percent.

The mean “third law” value of $\Delta H_{s}^{{}^{\circ}}(298)$ from table 4 is 134.9±1.0 kcal/mole, from which we estimate a normal boiling point of 4,100 ±100 °K by extrapolating Stull and Sinke’s tables. The least-squares line through the accepted data for the temperature range 1,916 °K to the melting point is given by:

$$\mathrm{Log}\;P_{atm}=6.761-\frac{27,575}{T}$$

or

$$\mathrm{Log}\;P_{mm}=9.642-\frac{27,575}{T}.$$

This line is drawn in figure 1.

### 3.2. Iridium

Iridium vapor pressures and the corresponding values of $\Delta H_{s}^{{}^{\circ}}(298)$ calculated from tabulated free energy functions [4] are given in table 4.

The iridium data had the best internal consistency, and this may have been a result of the greater purity of the sample. The results of the first four runs in table 4 were partially attributed to the cleanup of the sample and were rejected. Barely significant mass changes were recorded during the period of the fifth and eighth runs, and the results of these runs were also rejected.

The overall limits of error of the absolute value of $\Delta H_{s}^{{}^{\circ}}(298)$ were estimated by taking into account the scatter of the data, the uncertainty in the temperature (±25 °K), and the uncertainty in the weight-loss measurements. No allowance was made for the uncertainty in the free-energy functions or in the sublimation coefficient. The overall limits of error of the absolute value were estimated to be ±2.0 kcal/mole. The corresponding overall limits of error of the vapor pressures are about ±60 percent.

**Figure 2:**
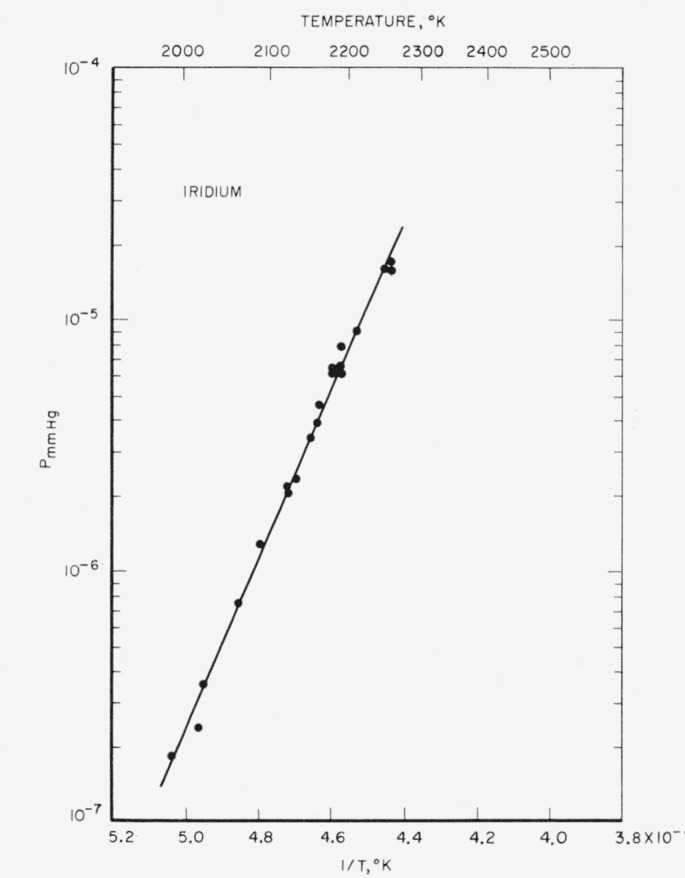
Vapor pressure of iridium versus reciprocal of absolute temperature.

In figure 2 the accepted experimental data are plotted, and the least-squares line for the data is shown. The equation of the line, which applies to the temperature range 1,986 to 2,260 °K, is:

$$\mathrm{Log}\;P_{\text{atm}}=7.139-\frac{33,337}{T}$$

or

$$\mathrm{Log}\;P_{\text{mm}}=10.020-\frac{33,337}{T}.$$

The mean $\Delta H_{s}^{{}^{\circ}}(298)$ is 159.9±2.0 kcal/mole from which a boiling point of 4,800±100 °K is estimated. These results are in agreement with those of Panish and Reif [10] within the limits of experimental error.

### 3.3. Rhodium

**Figure 3:**
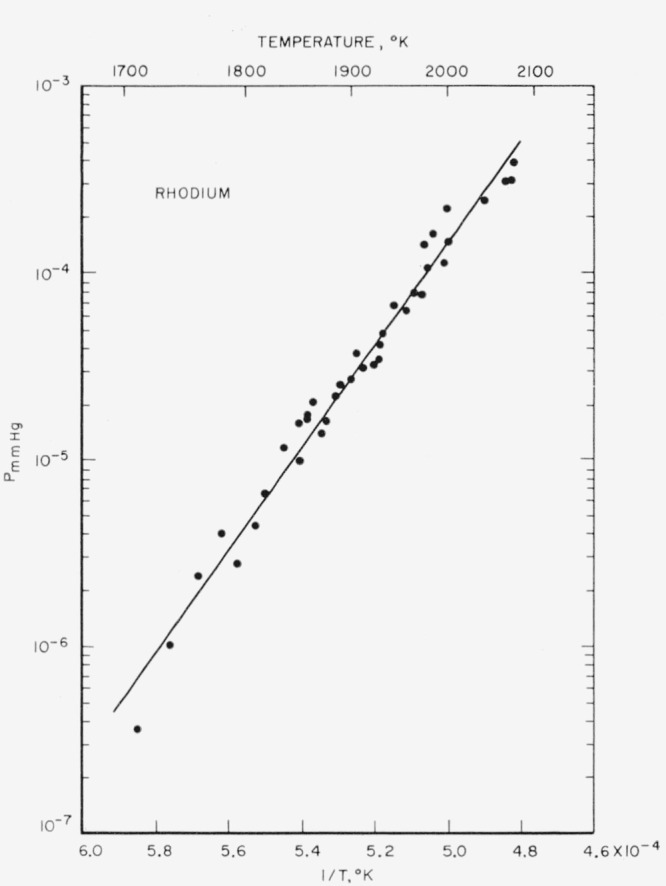
Vapor pressure of rhodium versus reciprocal of absolute temperature.

Rhodium vapor pressures and the heats of sublimation based on Stull and Sinke’s tabulated free energy functions [4] are given in table 5. The vapor pressures over the range 1,709 to 2,075 °K are plotted in figure 3, together with the least-squares line through the experimental points.

The rhodium data had more general scatter than the iridium; in particular, some of the earlier runs (see table 5) yielded lower heats of sublimation than were obtained towards the end of the sequence of runs, over the same temperature range. It is possible that the more volatile impurities in the sample were contributing to the scatter, but the deviations of the experimental points from their mean did not justify the rejection of any of the data. During a long sequence of runs on the same sample, the surface becomes thermally etched, leading to an increase in the emissivity. Such an effect would also contribute to an apparent increase in the heats of sublimation as the sequence progressed.

The mean $\Delta H_{s}^{{}^{\circ}}(298)$ is 132.5±2.0 kcal/mole from which a normal boiling point of 4,000±100 °K is estimated. The least squares fit to the data is given by:

$$\mathrm{Log}\;P_{\text{atm}}=6.894-\frac{27,276}{T}$$

or

$$\mathrm{Log}\;P_{\text{mm}}=9.775-\frac{27,276}{T}.$$

The mean heat of sublimation agrees with that of Panish and Reif within the limits of experimental error.

The overall limits of error of the absolute value of $\Delta H_{s}^{{}^{\circ}}(298)$ were estimated by taking into account the scatter of the data, the uncertainty in the temperature (±20 °K), and the uncertainty in the weight-loss measurements. No allowance was made for the uncertainty in the free-energy functions or in the sublimation coefficient. The overall limits of error of the absolute value were estimated to be ±2.0 kcal/mole. The corresponding overall limits of error of the vapor pressures are about ± 65 percent.

## Figures and Tables

**Table 1:** Summary of published data on volatilization of iridium and rhodium

Substance	Normal boiling point	$\Delta H_{s}^{{}^{\circ}}(298)$	Reference
	*°K*	*kcal/mole*	
Iridium	$\left\{ \begin{array}{l} 4673\\ 5573\\ 4800\\ 4400 \end{array}\right.$	…………	Mott [1].
		…………	Richardson [2].
		165	Brewer [3].
		150	Stull, Sinke [4].
Rhodium	$\left\{ \begin{array}{l} 4273\\ 4773\\ 4150\\ 4000 \end{array}\right.$	…………	Mott [1].
		…………	Richardson [2].
		138	Brewer [3].
		133	Stull, Sinke [4].

**Table 2:** Observations of melting point of platinum under experimental conditions (ϵ_λ_ = 0.308)

Brightness temperature	Thermodynamic temperature	Evidence of surface melting
*°C*	*°K*	
1567±5	2043	Not melted.
1575±5	2053	Melted.
1570±5	2047	Possibly melted slightly; evidence masked by preceding run.
1562±5	2037	Not melted.
1562±5	2037	Not melted.
1565±5	2041	Partially melted.
	2042±1	Accepted melting point of Pt.

**Table 3:** Vapor pressures and heats of sublimation of platinum

Temperature	Duration of run	Weight loss	Vapor pressure	$\Delta H_{s}^{{}^{\circ}}(298)$
*°K*	*min*	*μg*	*mm* Hg	*kcal/mole*
1710	130	37	1.68×10^−7^	*136.3
1822	36	103	1.74×10^−6^	*136.6
1764	85	…………	…………	…………
2043	10	1798	1.16×10^−4^	135.8
1990	2	214	6.85×10^−5^	134.4
1962	2	126	3.99×10^−5^	134.7
1926	3	92	1.93×10^−5^	135.0
1916	6	198	2.07×10^−5^	134.1
1917	25	75	1.88×10^−6^	*143.3
1909	30	23	4.74×10^−7^	*147.9
1917	28	20	4.44×10^−7^	*148.8
1842	29	…………	…………	…………
1955	4	215	3.41×10^−5^	134.8
1928	10	…………	…………	…………
1968	4	244	3.88×10^−5^	135.2
1951	5	…………	…………	…………
1980	2	158	5.03×10^−5^	134.9
1949	20	…………	…………	…………
1970	7	25	2.24×10^−6^	*146.5
1976	4.5	68	9.58×10^−6^	*141.2
1986	3.5	276	5.03×10^−5^	135.4
2000	2	231	7.39×10^−5^	134.8
2025	2	269	8.68×10^−5^	135.8
2034	2	374	1.21×10^−4^	135.0
2053	.5	174	2.25×10^−4^	**133.7
2047	1	267	1.73×10^−4^	134.4
2037	1	205	1.32×10^−4^	134.9
2037	1	206	1.33×10^−4^	134.8
2023	1	178	1.15×10^−4^	134.5
2016	1	170	1.09×10^−4^	134.3
2003	1	109	6.98×10^−5^	135.2
	
Mean $\Delta H_{s}^{{}^{\circ}}(298)$				134.9
Standard deviation				0.5

Data are presented in experimental sequence, a dashed entry indicating that no significant weight loss was detected during a run.

* Rejected.

** Sample partially melted; data rejected.

**Table 4:** Vapor pressures and heats of sublimation of iridium

Temperature**	Duration of run	Weight loss	Vapor pressure	$\Delta H_{s}^{{}^{\circ}}(298)$
*°K*	*min*	*μg*	*mm* Hg	*kcal/mole*
1964	1	60	3.98×10^−5^	*136.6
1990	1	11	7.11×10^−6^	*145.2
2029	1	4	2.94×10^−6^	*151.5
2059	1	1	3.28×10^−7^	*162.7
2134	1	4	3.00×10^−6^	*159.1
2185	1	9	6.08×10^−6^	159.8
2016	22	8	2.29×10^−7^	160.8
2077	75	3	3.07×10^−8^	*173.9
2190	12	108	6.26×10^−6^	160.1
2190	13	109	5.80×10^−6^	160.4
2260	9	192	1.50×10^−5^	161.1
2257	12	277	1.62×10^−5^	160.6
2211	9	110	8.48×10^−6^	160.2
2179	12	103	5.96×10^−6^	159.5
2249	8	172	1.51×10^−5^	160.3
2190	11	120	7.59×10^−6^	159.2
2178	10	88	6.11×10^−6^	159.3
2161	10	62	4.30×10^−6^	159.6
2150	30	142	3.26×10^−6^	160.0
2160	20	108	3.71×10^−6^	160.2
2130	40	131	2.23×10^−6^	160.1
2121	45	130	1.98×10^−6^	160.0
2120	42	127	2.06×10^−6^	159.7
2088	58	105	1.23×10^−6^	159.5
2060	50	54	7.28×10^−7^	159.5
2021	90	46	3.44×10^−7^	159.6
1986	180	48	1.78×10^−7^	159.5
	
Mean $\Delta H_{s}^{{}^{\circ}}(298)$				159.9
Standard deviation				0.5

Data are presented in experimental sequence.

* Rejected.

** Emissivity, *ϵ*_λ_, assumed to be 0.33 at λ=0.65*μ*.

**Table 5:** Vapor pressure and heats of sublimation of rhodium

Temperature**	Duration of run	Weight loss	Vapor pressure	$\Delta H_{s}^{{}^{\circ}}(298)$
*°K*	*min*	*μg*	*mm* Hg	*kcal/mole*
1708	25	90	3.15×10^−6^	*126.4
1774	80	82	9.16×10^−7^	*135.7
1851	40	417	9.43×10^−6^	132.9
1859	5	89	1.62×10^−5^	131.5
1851	6	101	1.52×10^−5^	131.1
1864	5	109	1.98×10^−5^	131.1
1942	3	205	6.32×10^−5^	131.9
1926	3	127	3.90×10^−5^	132.7
1921	4	133	3.06×10^−5^	133.3
1977	2	214	1.00×10^−4^	132.5
1966	2	157	7.30×10^−5^	133.0
1954	2.5	159	5.92×10^−5^	133.0
1930	2.5	122	4.50×10^−5^	132.4
1904	3	116	3.56×10^−5^	131.6
1900	3	83	2.53×10^−5^	132.6
1890	3	78	2.37×10^−5^	132.2
1857	3	54	1.64×10^−5^	131.3
1836	3	37	1.12×10^−5^	131.2
1819	4	28	6.34×10^−6^	132.1
1781	6	26	3.90×10^−6^	131.1
1760	12	31	2.32×10^−6^	131.4
1983	1	162	1.51×10^−4^	131.2
2001	1	224	2.11×10^−4^	131.1
1973	1	143	1.34×10^−4^	131.1
2071	.5	155	2.95×10^−4^	134.2
2065	1	307	2.92×10^−4^	133.8
2041	1	242	2.29×10^−4^	133.3
2075	1	383	3.65×10^−4^	133.5
1971	2	155	7.21×10^−5^	133.4
1927	2	71	3.26×10^−5^	133.5
1871	3	43	1.32×10^−5^	133.0
1809	5	24	4.28×10^−6^	132.8
1736	20	22	9.80×10^−7^	132.6
1794	9	27	2.69×10^−6^	133.3
2012	1	147	1.38×10^−4^	133.5
1997	1	114	1.07×10^−4^	133.5
1886	3	69	2.11×10^−5^	132.3
1910	3	98	2.99×10^−5^	132.7
1875	4	65	1.48×10^−5^	132.9
1709	100	40	3.48×10^−7^	134.1
	
Mean $\Delta H_{s}^{{}^{\circ}}(298)$				132.5
Standard deviation				1.0

Data are presented in experimental sequence.

* Rejected.

** Emissivity, *ϵ*_λ_. assumed to be 0.242 at λ=0.667*μ*.

## Footnotes

1 Figures in brackets indicate the literature references at the end of this paper.

2 It is often the case that the effects of *α* varying within the limits 0.5 and 1.0 would not be detected in the course of measurements, due to the imprecision of our knowledge of such factors as temperature differences.
